# Diagnostic Tools for the Identification of *Babesia* sp. in Persistently Infected Cattle

**DOI:** 10.3390/pathogens8030143

**Published:** 2019-09-09

**Authors:** J. Antonio Alvarez, Carmen Rojas, Julio V. Figueroa

**Affiliations:** Babesia Unit, CENID-Salud Animal e Inocuidad, INIFAP, 62550 Jiutepec, Mexico (J.A.A.) (C.R.)

**Keywords:** babesia, diagnosis, persistent infection

## Abstract

Bovine babesiosis is a tick-borne disease of cattle caused by the protozoan parasites of the genus *Babesia*. *Babesia bovis*, *Babesia bigemina* and *Babesia divergens* are considered by International health authorities (OIE) as the principal species of Babesia that cause bovine babesiosis. Animals that recover from a babesial primo infection may remain as persistent carriers with no clinical signs of disease and can be the source of infection for ticks that are able to acquire *Babesia* parasites from infected cattle and to transmit *Babesia* parasites to susceptible cattle. Several procedures that have been developed for parasite detection and diagnosis of this infectious carrier state constitute the basis for this review: A brief description of the direct microscopic detection of *Babesia*-infected erytrocytes; PCR-based diagnostic assays, which are very sensitive particularly in detecting *Babesia* in carrier cattle; in-vitro culture methods, used to demonstrate presence of carrier infections of *Babesia* sp.; animal inoculation, particularly for *B. divergens* isolation are discussed. Alternatively, persistently infected animals can be tested for specific antibabesial antibodies by using indirect serological assays. Serological procedures are not necessarily consistent in identifying persistently infected animals and have the disadvantage of presenting with cross reactions between antibodies to *Babesia* sp.

## 1. Introduction

Bovine babesiosis is a parasitic disease caused by an intraerythrocytic protozoan of the genus *Babesia*. The most important species that affect cattle are *Babesia bovis*, *Babesia bigemina* and *Babesia divergens* [1,2]. *B. bovis* and *B. bigemina* are currently present in Africa, Australia, Central and South America between 40° N and 32° S. *B. divergens* is distributed in North West Europe, Spain, Great Britain, Ireland and its main vector is *Ixodes ricinus* [1]. The vectors for *B. bovis* and *B. bigemina* are *Rhipicephalus* (*Boophilus*) *microplus*, *R. annulatus* and *R. geigyi*; *B. bigemina* is also transmitted by *Rhipicephalus evertsi* [3,4].

Throughout the clinical phase of the disease, general findings include fever above 41 °C, anemia, depression, anorexia, dehydration, hemoglobinuria and death of some affected animals. Nervous signs are regularly observed as result of sequestration of *B. bovis*-infected erythrocytes in cerebral capillaries. In addition, *B. divergens* has a particularly special importance as a dangerous zoonosis.

During the acute phase of the disease the parasites are microscopically detected in circulation, and parasitemias with less than 0.5% of *B. bovis*-infected erythrocytes (IE), 3% *B. bigemina*-IE, and 35–40% *B. divergens*-IE can be reached [1,3]. Animals that recover from clinical disease become persistently infected with *B. bovis* and/or *B. bigemina* with low levels of parasitemia. The persistence of the infection by *B. bovis* may remain for life [5]. *B. bigemina* infected animals remain as carriers for up to 22 months. Throughout the carrier stage a fluctuating *Babesia* parasitemia is observed, and tick infection occurs [6]. Epidemiologically, it is considered important to identify carrier animals, because they are associated with infection risks as they serve as reservoir of infection for both, ticks and naive cattle [7,8]. Occurrence of babesiosis depends on the presence of suitable vectors; however, a major risk of introducing *Babesia* parasites is through the importation of asymptomatic carrier animals, which has a negative impact on international cattle trade. Also, babesiosis should be recognized as a dangerous zoonosis specifically that caused by *B. divergens*.

Different techniques have been implemented to diagnose bovine babesiosis, usually the first choice is the thin or thick blood Giemsa-stained smear to microscopically demonstrate the presence of parasites as etiology of the clinical symptoms. To probe cattle exposure to *Babesia* or the passive transfer of antibodies by colostrum in calves, serological assays have been utilized, such as the Indirect Fluorescent Antibody Test (IFAT), Enzyme-Linked Immunosorbent Assay (ELISA) and Immunochromatography Test (ICT), as these tests provide information on the humoral immune response. These procedures very often are described as diagnostic tests lacking specificity, are time-consuming and difficult to perform. Additional disadvantages are the occurrence of cross-reactions and lack of discrimination between previous exposure and current infection [9]. Therefore, highly sensitive and specific molecular assays have been developed to detect *B. bovis, B. bigemina* and *B. divergens*. These are techniques that allow for the identification of persistent infection through the direct detection of the parasite genomic DNA by PCR assays (see Tables S1 and S2 for a summary of PCR assays that have been developed and utilized for cattle Babesia).

A series of molecular epidemiological surveys have been conducted in various countries to detect and genetically characterize *Babesia* parasites present in apparently healthy cattle [10,11,12,13]. Molecular methods based on the detection of *Babesia* DNA in blood by PCR techniques provides results with high sensitivity and specificity. Recently, nested PCR (nPCR) assays targeting *B. bovis* rhoptry-associated protein-1 (BbovRAP-1); *B. bovis* spherical body protein 2 (BboSBP2); *B. bovis* spherical body protein 4 (BboSBP4); *B. bigemina* Apical Membrane Antigen-1 (BbiAMA-1) and *B. bigemina* rhoptry-associated protein-1a (BbiRAP-1a) have proven to be a powerful tool for epidemiological investigations in apparently healthy, persistently infected cattle in Pakistan, Ghana, Mongolia, Brazil, Egypt, South Africa, Myanmar, Thailand, Syria, India, and the Philippines [10,14,15,16,17,18,19].

Cattle, as well as buffaloes, have been screened with highly sensitive PCR assays to detect persistently infected animals in order to genetically characterize the Merozoite Surface Antigen (MSA-1) diversity in *Babesia bovis* populations present in cattle premises from Mexico [20], Thailand, Brazil and Ghana [21], Vietnam [22], Thailand [23], Sri Lanka, Mongolia and Vietnam [24].

The importance of persistently infected animals is due to the fact that these can be the source of infection for animals that come from areas free of babesiosis that are introduced into herds where there are ticks and *Babesia* are endemic. The purpose of the review is to describe briefly the main diagnostic techniques, that allow for the identification of persistently infected cattle that remain as asymptomatic carriers of bovine babesiosis.

## 2. Direct Identification of *Babesia* sp.

### 2.1. Blood Smears

It is routinely the easiest way to detect parasites in the blood stream. Detection of parasites can be accomplished by using Giemsa or acridine orange-stained blood smears. The sensitivity of stained smears is low, thus false negatives are regularly observed [9]; this is common if the operator does not have the expertise to perform a good diagnosis [25]. The most important feature is that these methods are only helpful in detecting infected erythrocytes during the acute phase of the disease. Even in clinically affected animals pathogen detection becomes difficult because of the low number of parasites present in circulating blood, particularly on *B. bovis* infections [1]. Thus, parasites cannot be identified neither during the prepatent and convalescent stages, nor when animals are as asymptomatic carriers.

Blood films stained with Giemsa or with a fluorescent dye such as acridine orange, could favor the detection of parasites by microscopic examination using immersion oil, a 10x eyepiece and 100x objective lens. While this method shows low sensitivity [26], *B. bovis* can be recovered from capillary blood collected from the ear pinna or tail, whereas *B. bigemina* and *B. divergens* are usually found in venous blood samples. Out of a single suspicious animal, multiple thick and thin blood smears must be microscopically examined due of the low number of *Babesia bovis*-infected erythrocytes. In *B. divergens* infection, thin smears are preferred for visualizing the organisms. Morphologically, parasites appear as attaché small pyriform pairs with sizes ranging from 1.5 to 1.9 µm, and as ring stages from 1.5–1.8 µm. The disease usually progresses very fast, the parasitemia varies from 5–80%. Thus, parasites are easily detected by light microscopy [27,28,29]. While *B. bigemina* can be detected in the general circulation as relatively large round merozoite, with size varying from 2.3 µm, with an irregularly elongated shape with up to 5 µm in length, or as pear shaped forms in acute angle. *B. bovis* parasites appear as paired forms often in obtuse angle, measuring 1.5–2 µm in size and usually found in the middle of the erythrocyte; vacuolated ring forms are very common [29].

At necropsy, it is suitable to collect samples from brain, kidney, myocardium, liver and lung tissues. In *B. bovis*-infected animals, parasites in peripheral blood are usually present in parasitemia below 1% of infected erythrocytes; however, in imprints or impression smears made from brain tissue parasitemia are as high as 90% of the infected erythrocytes. This occurs because of the marked sequestration of infected erythrocytes in the microvascular beds of the brain, kidney and adrenal glands, particularly in splenectomized animals [30]. Cerebellar samples obtained through the occipital foramen have been utilized for microscopic detection of *B. bovis* in persistently infected cattle [31]. Nevertheless, it is not common to observe that asymptomatic carrier animals become fatal cases.

### 2.2. Polymerase Chain Reaction (PCR)

Babesial infections are difficult to detect because of the low number of parasites in peripheral blood. Therefore, DNA-based molecular methods have been developed with great advantages, such as high analytical sensitivity and specificity rates [32]. One of these molecular techniques is PCR, used to amplify, out of a single DNA fragment, millions of copies in vitro. The goal is to detect the presence or absence of a small DNA sequence. Advantages of the PCR assay include: It is more rapid as compared to in vitro cultivation of the parasite; it has high analytical and diagnostic sensitivity and specificity rates; Confirmation of the specificity of the assay is accomplished by sequencing the amplicons. However, sometimes PCR is not specific enough, so nested PCR should be carried out to reduce background to non-specific amplification of DNA. The most important disadvantages of the PCR assay are: PCR setting and running requires technical skills; cross-contamination risk is possible; PCR is not able to differentiate between living or dead parasites, and has a high cost for equipment, consumables and reagents.

Different PCR formats have been used, one of the first PCR assays performed was that developed for detection of *B. bigemina* [6]. The PCR-DNA assay coupled with a nonradioactive DNA probe was sensitive enough to detect cattle persistently infected with *B. bigemina*. Analytical sensitivity assays showed detection of as low as 100 fg of parasite genomic DNA equivalent to 0.0000001% of infected erythrocytes. The analytical specificity was demonstrated by lack of DNA amplification in reactions containing *B. bovis*, *A. marginale*, *S. aureus*, *S. typhimurium*, *E. coli*, *P. hemolytica*, *B. abortus*, *M. bovis* and bovine leukocyte DNA templates [6]. A colorimetric *B. bigemina* DNA probe was utilized in an epidemiological survey, which allowed to detect parasitemias as low as 0.001%. Thus, this assay turned out good to detect asymptomatic carriers in the field [33]. Similarly, detection of *B. bovis* infected carrier cattle was demonstrated by PCR amplification, by detecting an apocytochrome b gen. The sensitivity was about one infected erythrocyte per 0.5 mL of blood. This was highly specific for *B. bovis*, as no other hemoparasites were detected [34]. Additionally, both reports suggested that the specific PCR assays are broadly applicable for detection of parasite strains from different geographic regions. A multiplex PCR test coupled with a nonradioactive probe was used to detect, in the same blood sample, *B. bigemina*, *B. bovis* and *Anaplasma marginale*. The analytical sensitivity was 0.00001%, 0.00001%, 0.0001%, for *B. bigemina*, *B. bovis* and *A. marginale*, respectively. Persistent infection was confirmed in cattle as blood samples collected from cattle previously inoculated with *B. bigemina* (1 year), *B. bovis* (4 years), and *A. marginale* (2 years) were consistently detected with the PCR assay [7]. An improvement of the PCR system showed that the assay‘s analytical sensitivity allowed to detect parasitemias as low as 0.000001%, which represented around 3 *B. bovis*-infected erythrocytes contained in 20 µl of packed blood cells [35]. Once persistently infected animals are identified by PCR/DNA probe detection, there are changes in parasitemia levels, so that the parasite replication dynamics could have an adverse effect in the efficiency of the PCR assay [35,36]. A nested PCR assay also has been used for detection of *B. bovis* and *B. bigemina* not only in cattle (*Bos taurus*), but also in water buffaloes (*Bubalus bubalis*). Both types of animals have been detected as asymptomatic carriers [37,38]. It is also possible to perform DNA extraction from already stained blood smears, enabling an easier way to detect asymptomatic carriers in laboratory collection samples [39].

### 2.3. Real-Time PCR (RT-PCR)

This technique involves the analysis of the parasite genome by incorporating fluorogenic probes that release fluorescent signal during DNA amplification. The most important features over conventional PCR assays are the visualization without gel electrophoresis analysis, fast performance, closed automatized amplification, low risk of cross contamination and the quantitative results. To detect and measure the amount of the target DNA, a signal must be generated, which is proportional to the amount of amplified product in real time. This assay uses fluorescent technologies for detection and has different formats. In RT-PCR the overall assay requires less amount of the template material as compared to conventional PCR. Important disadvantages include that the equipment is too costly, higher expertise and technical skills are required for developing the assay.

Furthermore, the RT-PCR assay has high sensitivity, specificity and reproducibility, also is much more sensitive than microscopic analysis of Giemsa-stained blood smears. These features favor its use for diagnosis and quantification of persistently infected animals. In experimental and field surveys, a quantitative PCR (qPCR) developed as a TaqMan assay was instrumental as a duplex format for the diagnosis of *B. bovis* and *B. bigemina*. In addition, *B. divergens* was detected by a fluorescence resonance energy transfer (FRET) probe, which allowed to differentiate among *B. bovis*, *B. bigemina* and *B. divergens* [40]. By using the RT-PCR assay, *Babesia* infection has been detected in blood samples at 1000-fold lower concentrations as compared to the standard PCR assays [41]. Okino et al., [42] verified that *B. bovis* infected calves were better detected by qPCR than by direct microscopy (100% vs. 10% positivity, respectively). Nowadays, advances in qPCR have shown better sensitivity and specificity, less variability to offer excellent validity and reliability in the identification of persistently infected cattle.

### 2.4. Loop-Mediated Isothermal Amplification (LAM)

LAMP is a commonly used technique, it is a rapid, cheap, highly sensitive and specific assay, which relies on the auto-cycling strand displacement synthesis of target DNA by Bst DNA polymerase under isothermal conditions. This assay allows detection and discriminates between *B. bovis* and *B. bigemina* species. The analytical sensitivity of LAM is 0.1 pg DNA for both species’ assays [43]. A multiplex loop-mediated isothermal amplification (mLAMP) was developed; in this assay, primers for rhoptry-associated protein-1 genes of *B. bovis* and *B. bigemina* were designed, a restriction enzyme cleavage site was inserted into the two pairs of primers for each species, and the combination of eight total primers allowed for the development of the mLAMP method which is useful for the simultaneous detection of *B. bovis* and *B. bigemina* [44].

Improvements made for to this method, include the avoidance of gel electrophoresis for visualization of LAMP products, as the chromatographic lateral flow dipstick (LAMP-LFP) format has been applied to reveal products in a simpler and faster way. LAMP-LFP included a set of four primers targeting and amplifying six distinct regions of the *Babesia* sp. cytochrome b gene under isothermal conditions. This procedure was able to detect 0.85 fg and 0.14 fg of *B. bigemina* and *B. bovis*, respectively, which represents 100-fold higher analytical sensitivity than a conventional PCR assay. This assay could be utilized to identify persistently infected carrier animals with very low parasitemias [45]. LAMP is considered a simple diagnostic method, which does not require special equipment, is fast and presents a low risk of contamination. The assay may be used for screening large number of samples in the field. Therefore, it has a promising perspective as a tool for the molecular diagnosis of bovine babesiosis.

### 2.5. Subinoculation

Parasite detection in infected animals having low parasitemia can also be made by xenodiagnoses. Thus, detection of carrier or persistently infected cattle may be accomplished by the subinoculation of infected blood into splenectomized calves for *B. bovis* and *B. bigemina* isolation, or into gerbils for *B. divergens* isolation to determine the presence of *Babesia* sp. viable parasites in the susceptible recipient animals [27,46]. The Mongolian gerbil (*Meriones unguiculatus*) is an excellent laboratory animal model to infect with *B. divergens*. In contrast to bovines, inoculated recipient gerbils show acute and very often fatal cases of babesiosis [47]. However, animal inoculation is not suitable for diagnostic purposes, as the procedure can take weeks, so it is discouraged if other methods are available. Probably xenodiagnoses, followed by molecular detection at some point post-inoculation, could be a more reliable procedure as viable parasites and DNA even at very low parasitemia could be detected [27,46].

### 2.6. In Vitro Culture of Babesia sp.

Different *Babesia* sp. have been adapted to continuous cultivation in vitro, originally described using an agitated culture flask technique. In vitro cultivation of *Babesia* basically consists of a red blood cell suspension from a bovine donor and a chemically defined culture medium supplemented with 40% adult bovine serum [48]. General features of this technique include a suspension of red blood which are added to give a cell volume of 10–15%, commercial culture medium, bovine serum as medium supplement (40%) at a defined pH (7.2–7.4), in a 5% O_2_^−^ 95% air atmosphere, in which the cultures flasks are kept at 37.5 °C. Different types of culture medium have been utilized (M-199, RPMI 1640, NCT-135) [49]. Later, continuous in vitro growth of *B. bovis* was performed by using similar components but changing to a stationary layer of erythrocytes rather than a cell suspension calling this procedure as the microaerophilous stationary phase (MASP) system.

Also, it was recently demonstrated that advanced DMEM/F12 medium with a mixture of insulin-transferrin-selenite (M-ITS) is able to support proliferation of *B. bovis* and *B. bigemina* in a culture medium free of bovine serum [50,51]. Thus, isolation of *B. bovis*, *B. bigemina* or *B. divergens* by in vitro cultivation has been proposed as an alternative method for diagnosis or detection of persistent infection in cattle [50]. *B. divergens* has been recovered from different isolates by in vitro cultivation up to 9 months after clinical manifestation of disease in naturally infected animals; *B. divergens* parasites were well adapted to in vitro cultivation during several subcultures, after which they were cryopreserved and resuscitated [52]. *B. divergens* was originally cultivated in RPMI medium supplemented with 10% human serum, then serum was removed and after a period of adaptation the culture was continuously grown reaching up to 3% of parasitized erythrocytes [53]. Parasites have also been successfully cultured by using calf sera instead of adult bovine sera [54]. More recently *B. divergens* and *B. bigemina* species were cultured for a short-term using serum-free GIT medium [55].

Isolation of field *B. bigemina* strains was described from engorged *R.* (*Boophilus*) *microplus* female ticks that were collected from cattle suffering clinical babesiosis [56]. Also, isolation of *B. bovis* has been described through in vitro culture of blood from an asymptomatic carrier [50]. *Babesia* spp. are routinely cultured in vitro for different study purposes such as diagnosis, immunity, and identification of proteins associated to the invasion process, pathophysiology, and chemotherapy of babesiosis as well as to produce *B*. *bovis* to sequence its genome [57].

Parasite isolation by the in vitro culture procedure is not routinely used as tool in detecting persistently infected cattle, most probably because it is not affordable to diagnostic laboratories, takes a variable time for isolation and technical expertise is required to grow *Babesia* parasites, remaining mostly as a useful resource tool for research laboratories.

### 2.7. Severe Combined Immunodeficiency (SCID) Mice

An interesting SCID mice model harboring circulating bovine red blood cells has been used for *Babesia* parasites isolation. By using this procedure, initial blood samples were obtained from grazing calves and inoculated into SCID mice, which not only showed high level of parasitemia, but had clinical symptoms characterized by haemoglobinuria and hemolytic anemia. Some mice even exhibited nervous signs and parasites were isolated from them. In this experiment, SCID mice were prepared by intravenously transfusing splenectomized animals with bovine red blood cells (BoRBC) twice. A mouse anti-RBC monoclonal antibody was also added to achieve rapid replacement of circulating RBCs with BoRBC [58]. Other studies performed also describe the use of the SCID mice as a model to infect with *B. bovis*, developing high level of parasitemia and nervous symptoms and pathology, such as that occurring in infected cattle [59,60]. The SCID-mice model transfused with human erythrocytes has also been used to isolate and detect *B. microti*-like parasites [61].

### 2.8. Detection of Babesia in Ticks

The life cycle of *B. bovis* and *B. bigemina* in the vector includes sporokinetes (kinetes) which infect tick tissues through the hemolymph. *Babesia* parasites can be identified in *Rhipicephalus (Boophilus) microplus* engorged females by light microcopy of tick-hemolymph searching for kinetes [62,63]. It has been demonstrated that female ticks fed to repletion on persistently infected calves are able to produce larval progeny with *B. bovis* infection [64]. Thus, it is important to consider that ticks are directly associated to cattle persistently infected with *Babesia*. On the other hand, climate change is increasing the risk of transmission by altering the distribution of ticks in the field [46].

For the detection of *Babesia* in both ticks and cattle, DNA probe-based techniques were developed and used [65]. Another highly sensitive analysis of PCR with a subsequent sequencing of the amplicons revealed an infection with *B. divergens*-like parasites in nymphs and adult ticks *Ixodes ricinus* [66]. Using a nested PCR assay *B. bigemina* was detected in females with *B. microplus* fed with cattle raised in an area with endemic babesiosis [67]. By using light microscopy, *B. bovis* and *B. bigemina* are detected in hemolymph of engorged female and squashes of eggs from *B. microplus* collected out of *Bos taurus* x *Bos indicus* dairy cattle located in a tropical area [67]. However, in a study that quantified the passage of *B. bovis* through multiple stages of tick development, from acquisition in the acute infection by female ticks to the end of the transmission by infected larval progeny, it was demonstrated that tick hemolymph infection sometimes is undetectable, but transmission to larval progeny occurs as demonstrated in a tick larvae-infested bovine. This suggests that ticks derived from cattle persistently infected are competent to passing parasites to their larval offspring [64]. It is known that once engorged, not all the *Rhipicephalus (Boophilus) microplus* ticks that fed on cattle become infected; however, it has been demonstrated that cattle infested with *B. bigemina*-infected ticks are more infective for other ticks, than those infected by blood inoculation [68]. *Babesia* parasites have developed strategies to adapt to the feeding and molting of their tick vectors [69]. As hard ticks feed only once per instar, *Babesia* parasites have developed the ability to persist by successive tick developmental stages (transstadial transmission). Another strategy of *Babesia* persistence is transovarial transmission, allowing for the spread of the parasite from a single maternal tick to thousands of offspring. Since *R.* (*Boophilus*) *microplus* is the vector for *B. bovis* and *B. bigemina*, and *I. ricinus* for *B. divergens*; it is important to keep in mind that sexual reproduction of *Babesia* parasites occurs in ticks. Therefore, by detecting *Babesia* in ticks, the presence of persistently infected bovine can be inferred in a herd.

## 3. Indirect Immunologic Assays

In cattle reared in babesiosis endemic areas parasites are present in the blood stream at very low numbers, below the threshold of direct detection techniques. Thus, indirect serological methods such as the Indirect Fluorescent Antibodies Test (IFAT), Enzyme-Linked Immunosorbent Assay (ELISA) and the Complement Fixation Test (CFT) are used routinely. These tests have as main disadvantage that high antibody titers are not necessarily proving the presence of parasite infection, nor the protective immunity. In addition, false negative animals can be determined even in the presence of circulating parasites or in protected animals by sterile immunity [46].

Serological procedures are not necessarily consistent in identifying persistently infected animals and have the disadvantage of presenting with cross reactions between antibodies to *B. bovis* and *B. bigemina*. Overall, by using serological assays it is not possible to distinguish between past exposure and present infections, as antibodies usually persist by variable periods of time even in *B. bovis*, *B. bigemina* or *B. divergens*-cleared animals. In enzootically stable areas of bovine babesiosis, the clinical cases are absent along the year, but most of the cattle are *Babesia*-infected. Under those conditions, the cows have high antibodies titers that are able to transfer passively to calves through colostrum. Due to that, calves appear as seropositive, but in fact are false positive in terms of *Babesia*-infection [70].

### 3.1. Indirect Fluorescent Antibody Test (IFAT)

This test has been used extensively, consists in the combination of the high sensitivity of florescence microscopy with the strict specificity associated with immunological methods. A great advantage is the antigen production derived from *B. bovis*, *B. bigemina* and *B. divergens* cultured in vitro. It has been used widely, but cross reaction between *B. bovis* and *B. bigemina* is a major drawback for species-specific diagnosis [71]. The results are influenced by the operator subjective judgment, while the IFAT provides adequate sensitivity and it is easy to perform, its reduced specificity makes it non-viable in some laboratories and is not routinely followed nowadays. A high proportion of cattle infected with *B. divergens* did not have antibodies detectable by IFAT [25].

### 3.2. Enzyme-Linked Immunosorbent Assay (ELISA)

Originally, the ELISA was implemented by using crude merozoite lysate antigens from infected erythrocytes. Later, different versions of ELISAs were implemented to detect antibodies to *B. bovis*, *B. bigemina* and *B. divergens*. A competitive Enzyme-linked immunosorbent assay (cELISA), based on the ability of serum antibody to inhibit a monoclonal antibody (Mab) binding was instrumented. A purified recombinant *B. bovis* RAP-1 C-terminal construct was used as antigen, and the inhibition of binding of MAb to RAP-1 specific epitope by serum antibodies was measured to detect positive cattle [72].

The technique was improved to avoid cross-reactivity to *B. bigemina*-infected bovine sera [73]. Similarly, the assay was set up by using an epitope of *B. bigemina* within the C terminus of RAP-1. The test applied in an area with 75% prevalence showed high positive and negative predictive values, 100% and 95.9%, respectively [5]. Both assays favor the detection of early infections and apparently the long-term carrier stage. Recently, an indirect-ELISA was reported by using a chimerical multi-antigen, comprising gene fragments with B and T epitopes of three *B. bovis* antigens: MSA-2c, RAP-1 and the Heat Shock protein 20. The sensitivity and specificity rates reported were 95.9% and 94.3%, respectively. However, cross reactions were observed when samples from *B. bigemina* infected cattle were analyzed, thus suggesting that using this assay is an alternative test to detect anti-*Babesia* sp. antibodies [74]. In general, ELISA has replaced the IFAT as the preferred diagnostic serological test in a diagnostic laboratory, particularly because of the ease and objectivity in interpretation, as well as for the capacity for automatization to process a higher number of samples in a working day.

### 3.3. Complement Fixation Test (CFT)

A crude suspension of parasites was described as useful to implement a sensitive CFT to detect antibodies to *B. argentina* (*B. bovis*) in serum [75]. The CFT reaction is dependent on the presence of complement fixing antibodies, wherein most of the important isotype is the IgM immunoglobulin. Thus, early infections are detected, but it is not suitable for detecting chronically infected animals [76]. CFT was compared to the IFAT in different studies in cattle samples collected in the field. Both tests were able to detect and differentiate *B. bovis* and *B. bigemina* exposed cattle. The IFAT showed some advantages over the CFT such as earlier detection of antibodies, simplicity, economy and less consuming time [77]. Also, a rapid conglutination test (RCT) was compared to ELISA and IFAT with high concordance results [78]. This test showed high sensitivity and specificity rates, 90.9% and 97.6%, respectively. Thus, the RCT assay was considered as a sensitive, economical and easy assay, appropriate for conducting epidemiological surveys [76]. However, the RCT was not reported any more as a diagnostic tool for *B. bovis*. Other tests used in the past included the dot ELISA, the slide ELISA, the latex and card agglutination tests, however, none of these tests have been adopted for routine diagnostic use in laboratories around the world.

### 3.4. Immunochromatography

Serological tests have several restrictions for the diagnosis of bovine babesiosis, as laboratory materials, appropriate equipment and trained personnel are usually required, in addition to being labor intensive and time-consuming. Those limitations apparently are solved in part by the development of the immunochromatographic test (ICT), also known as lateral flow test or strip test. The ICT is a nitrocellulose membrane-based immunoassay which does not require any instrument, is rapid and sensitive. In addition, it has a great advantage for use in clinical and field application directly in the farms. An evident advantage is that results are provided in less than 15 min [79].

An ICT was described to detect antibodies to *B. bovis* and *B. bigemina* in serum samples from infected cattle, based on *B. bovis* recombinant merozoite surface antigen (rMSA-2c) and the recombinant C-terminal portion of the *B.* *bigemina* rhoptry-associated protein-1 (rRAP1/CT). The concordance rates for the ICT to detect antibodies to *B. bovis* were 92.3% and 90.3% as compared to the ELISA and IFAT assays, respectively; whereas the concordance rates to detect *B. bigemina* antibodies for both assays was 96.8% and 92.5%, respectively, and no detection of antibody cross-reactivity was reported [80].

Another study, using an ICT to detect antibodies to *B. bovis* (boICT) or *B. bigemina* (biICT) both individually and in a dual ICT using serum from bovine samples collected in the field, showed a kappa coefficient >0.7 when compared to the ELISA [81].

### 3.5. Alternative Methods

A microfluidic device to perform cell detection and separation with high resolution and specificity, based on impedance spectroscopy of *B. bovis*-infected erythrocytes was reported [82]. A feature to remark for this assay is that whole blood samples are added without any previous preparation, as the dielectric properties of leukocytes or platelets are different to those for the parasitized red blood cells. However, this biosensor shows also some limitations to detect parasitemias above 1%. Nonetheless, this device has been proposed for preparing normal and *B. bovis*-infected erythrocyte populations. In addition, it could be useful for the enrichment of *Babesia* sp. infected cells in small volumes of sample and has potential to be used in the diagnostics of babesiosis in a near future [83].

Flow cytometry is another technique that allows for the analysis of various parasite stages and phases of *Babesia divergens* cycle in its host erythrocytes, and the quantification of the variations in parasitemia. Considering that the intra-erythrocytic life cycle is fairly conserved with other species, the protocols can be utilized for different comparative analysis [84,85]. In addition, by using a fluorescent-activated cell sorter *B. bovis* parasites were separated and biologically cloned to study the virulence phenotype of the parasite populations [86,87]. Whether these types of alternative methods are able to detect low *Babesia* parasitemia in persistently infected cattle remains to be tested, as pathogen persistence in the host is an important strategy for successful pathogen transmission to ticks and for developing resistance against reinfection of hosts [88,89,90,91].

## 4. Conclusions

Cattle raised in endemic areas are apparently healthy despite being infected, indicating persistent infection with *Babesia*. *Rhipicephalus* ticks can acquire *Babesia* parasites from infected cattle even in the absence of detectable parasitemia. Although infection may be undetectable in bovine and tick samples, ticks are able to transmit *Babesia* parasites to susceptible cattle. These features have important implications for the understanding of *Babesia* transmission by *Rhipicephalus* ticks in bovine babesiosis endemic regions. Parasite detection and diagnosis of this infectious carrier state is therefore highly relevant for epidemiological purposes in terms of prediction and prevention of bovine babesiosis outbreaks and cattle trade. Out of the numerous tests that have been developed for diagnostic as well as epidemiological purposes this review focuses and briefly describes the direct detection methods that include light and fluorescence microscopy; PCR-based diagnostic assays turned out to be very sensitive particularly in detecting *Babesia* in carrier cattle; in-vitro culture methods and animal sub-inoculation, while cumbersome and time-consuming, can be used in a suitable laboratory and animal handling facilities to demonstrate presence of carrier infections of *Babesia* sp. and parasite isolation. Alternatively, persistently infected animals can be tested for specific antibabesial antibodies with the indirect serological assays. Serological procedures are not necessarily consistent in identifying persistently infected animals and have the disadvantage of presenting with cross reactions between antibodies to *Babesia* sp. Thus, it is not possible to distinguish between past exposure and present infections, as antibodies usually persist by variable periods of time even in *B. bovis*, *B. bigemina* or *B. divergens*-cleared animals.

## Supplementary Materials

The following are available online at https://www.mdpi.com/2076-0817/8/3/143/s1, Table S1: Primers for the PCR assays for *B. bigemina* reviewed in this study. Table S2: Primers for the PCR assays for *B. bovis* reviewed in this study.
