# Supplementary material for: Diagnostic Tools for the Identification of Babesia sp. in Persistently Infected Cattle
Source: Pathogens. 2019 Sep 9;8(3):143. doi: 10.3390/pathogens8030143 (PMC6789608; doi:10.3390/pathogens8030143)
Supplement: Supplementary file 1 [file pathogens-08-00143-s001.pdf]

Table S1. Primers for the PCR assays for *B. bigemina* reviewed in this study.

Species	Primer 5′ – 3′	Sequence	Product length (Bp)	Target gene (Genbank No.)	Reference
		Standard PCR		(S45366.1)	
	BiIA	CATCTAATTTCTCTCCATACCCCTCC	278	6–31	6, 36, 65
	BiIB	CCTCGGCTTCAACTCTGATGCCAAAG		257–283	
		Nested PCR			
	BilAN	CGCAAGCCCAGCACGCCCCGGTGC	170	61–83	
	BilBN	CCGACCTGGATAGGCTGTGTGATG		206–230	
		Standard PCR		SS rRNA (X59604)	
	GAU5	TGGCGGCGTTTATTAGTTCG	1,124 bp	409-428	10
B. bigemina	GAU6	CCACGCTTGAAGCACAGGA		1532-1515	
		Standard PCR		AMA-1 (AB481200)	
	BI-AMA-FI	TACTGTGACGAGGACGGATC	211 bp	745–764	11, 12, 19
	BI-AMA-RI	CCTCAAAAGCAGATTCGAGT		936–955	
		Standard PCR		AMA-1 (AB481200)	
		GTATCAGCCGCCGACCTCCGTAAGT			
	BI-AMA-F0	GGCGTCAGACTCCAACGGGGAACCG	738 bp	556–580	15, 16, 17
	BI-AMA-R0			1269–1293	
		Nested PCR			
	BI-AMA-FI	TACTGTGACGAGGACGGATC		745–764	
	BI-AMA-RI	CCTCAAAAGCAGATTCGAGT	211 bp	936-955	

			RAP-1α (M60879)	
	Standard PCR			
F	GAGTCTGCCAAATCCTTAC	879	ND	
R	TCCTCTACAGCTGCTTCG			16, 19
	Nested PCR			
F	AGCTTGCTTTCACAACTCGCC	412	ND	
R	TTGGTGCTTTGACCGACGACAT			

ND= Not Defined

Table S2. Primers for the PCR assays for *B. bovis* reviewed in this study.

Species	Primer 5′ – 3′	Sequence	Product length (Bp)	Target gene (Genbank No.)	Reference
Babesia bovis				RAP-1 (M38218.1)	
		Standard PCR			
	BoF	CACGAGGAAGGAACTACCGATGTTGA	356	656–681	7, 11, 12, 35, 65
	BoR	CCAAGGAGCTTCAACGTACGAGGTCA		990–1015	
		Nested PCR			
	BoFN	TCAACAAGGTACTCTATATGGCTACC	291	690–715	
	BoRN	CTACCGAGCAGAACCTTCTTCACCAT		962–987	
				SSrRNA (L31922)	
	JD127	TTGGCATGGGGGCGACCTTCACCCTCGCCC	275	428–457	8
	JD111	CCAAAGTCAACCAACGGTACGACAGGGTCA		670–699	
				SS rRNA (L31922)	
	GAU9	CTGTCGTACCGTTGGTTGAC		409-428	10
	GAU10	CGCACGGACGGAGACCGA	541 bp	1215-1198	

			SBP-2 (XM_001611726)	
	Standard PCR			
F	CCGAATTCCTGGAAGTGGATCTCATGCAACC	1236	ND	14
R	ATCTCGAGTCACGAGCACTCTACGGCTTTGCAG			
	Nested PCR			
F1	CGAATCTAGGCATATAAGGCAT	580	ND	
R1	ATCCCCTCCTAAGGTTGGCTAC			
			SBP-4 (AB594813)	
	Standard PCR			
F	AGTTGTTGGAGGAGGCTAAT	907	ND	16, 19
R	TCCTTCTCGGCGTCCTTTTC			
	Nested PCR			
F1	GAAATCCCTGTTCCAGAG	503	ND	
R1	TCGTTGATAACACTGCAA			

ND= Not Defined.
